# The Drosophila Helicase Maleless (MLE) is Implicated in Functions Distinct From its Role in Dosage Compensation*[Fn FN2]

**DOI:** 10.1074/mcp.M114.040667

**Published:** 2015-03-16

**Authors:** Simona Cugusi, Satish Kallappagoudar, Huiping Ling, John C. Lucchesi

**Affiliations:** From the ‡Department of Biology, Emory University, Atlanta, Georgia 30322

## Abstract

Helicases are ubiquitous enzymes that unwind or remodel single or double-stranded nucleic acids, and that participate in a vast array of metabolic pathways. The ATP-dependent DEXH-box RNA/DNA helicase MLE was first identified as a core member of the chromatin remodeling MSL complex, responsible for dosage compensation in Drosophila males. Although this complex does not assemble in females, MLE is present. Given the multiplicity of functions attributed to its mammalian ortholog RNA helicase A, we have carried out an analysis for the purpose of determining whether MLE displays the same diversity. We have identified a number of different proteins that associate with MLE, implicating its role in specific pathways. We have documented this association in selected examples that include the spliceosome complex, heterogeneous Nuclear Ribonucleoproteins involved in RNA Processing and in Heterochromatin Protein 1 deposition, and the NuRD complex.

Helicases are involved in all cellular transactions related to nucleic acid function and metabolism in prokaryotes and eukaryotes. These enzymes participate in transcription, RNA splicing, the stability of transcripts, and translation initiation, as well as in DNA-replication, repair, and recombination. Helicases use the energy produced by the hydrolysis of nucleotide triphosphates to catalyze the unwinding of DNA, RNA, and RNA:DNA hybrid molecules. They are also involved in protein displacement from RNA, RNA clamping, strand annealing, and RNA structure conversion (1). Eukaryotic helicases can be divided into two superfamilies, SF1 and SF2, which include three and nine families, respectively. Members of the different families can be distinguished on the basis of whether they use the hydrolysis of ATP or some other nucleotide triphosphates for energy release, whether they unwind DNA or RNA, and whether they require a 3′ overhang or a 5′ overhang to carry out their unwinding functions (2, 3).

The Drosophila helicase maleless (MLE)^1^ is a subunit of the MSL (male-specific lethal) complex that is responsible for dosage compensation - the regulatory mechanism involved in equalizing the levels of X chromosome-linked gene products between the sexes. In addition to MLE, the MSL complex contains an ubiquitin ligase (MSL2), a histone acetyl transferase (MOF), two structural proteins (MSL1 and MSL3), and one of two long non-coding RNAs (roX1 and roX2) that are necessary for its assembly and targeting. MLE is an ATP-dependent DEXH-box RNA/DNA helicase that *in vitro* prefers hybrid RNA:DNA or double-stranded RNA substrates with a short 3′ overhang (4). MLE exhibits some similarity to the ATPases present in complexes that remodel chromatin by altering the positioning or the architectural relationship between histone octamers and DNA. In contrast to MLE, none of these enzymatic subunits have been shown to possess double-stranded nucleic acid unwinding activity. The absence or loss of function of MLE leads to failures in assembly and targeting of the MSL complex to its numerous sites of action along the X chromosome in males, although MSL1 and MSL2 are present at a few “high affinity” or “entry” sites (5–10). Recent biochemical evidence indicates that assembly of the complex is initiated when MLE associates with a roX RNA and remodels its secondary structure allowing the binding of MSL2 and providing the core for the full recruitment of the other MSL subunits (11, 12).

Several considerations have led us to ask whether MLE participates in molecular events or pathways unrelated to dosage compensation: the MLE protein is present in the somatic cells of both males and females, and a mutation of *mle* (*mle^napts^*, where *napts* stands for *no action potential, temperature-sensitive*), originally isolated on the basis of its paralytic phenotype (13), exerts its effect in both males and females by preventing the remodeling necessary for appropriate splicing of the para mRNA (14). Furthermore, the mammalian ortholog of MLE, RNA helicase A (RHA/DHX9) (15) is involved in numerous and diverse functional interactions, from facilitating RISC assembly (16, 17) to translation initiation (18–20), or acting as a DNA-binding partner for EGFR-mediated transcription (21). As an initial approach we have carried out a mass spectrometry analysis of all of the proteins that co-immunoprecipitate with MLE in Drosophila S2 cells, when the MSL complex is present or when it is abrogated by RNA interference. We used S2 cells in order to determine the possible relationship of the MLE fraction associated with the MSL complex with the fraction involved in other functions. We also determined whether the co-immunoprecipitation depends on the presence of RNA. We believe that the study of the role that MLE plays in selected pathways in Drosophila will be of major use in understanding the function of RNA helicase A in similar pathways in humans. Here, we document the interaction of a selected group of proteins with MLE in order to provide preliminary evidence for its involvement in diverse regulatory pathways.

## EXPERIMENTAL PROCEDURES

### 

#### 

##### S2 Cells Transfection and RNAi Treatment

Transfections were performed as described in Yokoyama *et al.* (22). One to 3 days prior to transfection, S2 cells were treated with 10 μg/ml of MSL2 double-stranded RNA or GFP dsRNA. Additional dsRNA was added to maintain the 10 μg/ml concentration throughout the experiment. dsRNA was made following Ambion's MEGAscript protocol (Thermo Fisher Scientific, Waltham, MA). A list of the primers used can be found in Cugusi *et al.* (23).

##### Immunoprecipitation and Sample Preparation

S2 cells were transfected with a CuSO4-inducible FLAG-MLE plasmid, with a FLAG-GFP plasmid (24), or with the empty vector pMK33-C-FLAG-HA (a gift from S. Artavanis-Tsakonas). Three days after induction the cells were collected and lysed in ice for two hours in the following buffer: 150 mm NaCl, 50 mm Tris-HCl pH 7.5, Triton X-100 1%, and complete protease inhibitor mixture (Roche, Basel, Switzerland). The lysates were kept an additional hour at room temperature with or without 200 μg/ml of RNase A (Qiagen, Venlo, The Netherlands). 6 to 10 mg of protein extracts were incubated overnight at 4 °C with anti-FLAG M2 agarose beads (Sigma-Aldrich, St. Louis, MO), previously equilibrated in lysis buffer. In the mock sample 160 μg/ml of FLAG peptide (Sigma) were added to untransfected cells extracts during the incubation with the beads. Complexes were collected by centrifugation and washed four times in PBS buffer containing 1% Tween-20, and eluted for 1 h at 4 °C in 300 μl of PBS buffer containing 160 μg/ml FLAG peptide and protease inhibitor. Each sample was allowed to enter a SDS-PAGE gel, the gel was run briefly, and the bands containing the samples were cut out while they were still in the stacking part of the gel. Three 1D gel bands/sample were in-gel digested separately using the published protocol (25) and pooled. Pooled samples were dried in a speedvac and dissolved in 42 μl of 5% Formic acid. Samples of 40 μl were analyzed by LC/MS/MS.

##### Mass Spectrometry

Protein digests were separated using liquid chromatography with an NanoAcquity UPLC system (Waters, Milford, MA), then delivered to an LTQ Velos dual pressure linear ion trap mass spectrometer (Thermo Fisher) using electrospray ionization with an Captive Spray Source (Bruker, Billerica, MA) fitted with a 20 μm taper spray tip and 1.0 kV source voltage. Xcalibur version 2.1 was used to control the system. Samples were applied at 15 μl/min to a Symmetry C18 trap cartridge (Waters) for 10 min, then switched onto a 75 μm x 250 mm NanoAcquity BEH 130 C18 column with 1.7 μm particles (Waters) using mobile phases water (A) and acetonitrile (B) containing 0.1% formic acid, 7–30% acetonitrile gradient over 106 min, and 300 nL/min flow rate. A normalized collision energy of 30 was used. Data-dependent collection of MS/MS spectra used the dynamic exclusion feature of the instrument's control software (repeat count equal to 1, exclusion list size of 500, exclusion duration of 30 s, and exclusion mass width of −1 to +4) to obtain MS/MS spectra of the ten most abundant parent ions (minimum signal of 5000) following each survey scan from *m*/*z* 400–1400. The tune file was configured with no averaging of microscans, a maximum inject time of 200 msec, and automatic gain control targets of 3 × 10^4^ in MS1 mode and 1 × 10^4^ in MS2 mode.

##### Mass Spectrometry Data Analysis

An nr_20120621_fruitfly_7227_both.fasta database was used. This database was created by using protein sequences from *Drosophila melanogaster*, Taxon identifier 7227 (19577 protein sequences). We used reversed databases to estimate error thresholds (26). The database sequences and their reversed sequences were appended to 179 common contaminant sequences and their reversed forms for a final database of 39512 protein sequences. The database processing was performed with python scripts available at http://www.ProteomicAnalysisWorkbench.com.

RAW data from the mass spectrometer were converted to DTA files representing individual MS2 spectra using extract_msn.exe (version 5.0; Thermo Fisher). The group scan minimum count was 1, a minimum of 25 ions were required, the mass tolerance for combining DTAs was set to a very small value (0.0001 Da) so that spectra would not be combined, an absolute intensity of greater than 500 was required, and MH+ values had to be in the range of 550 to 4000 Da. SEQUEST (version 28, revision 12, Thermo Electron) searches for both sets of samples were performed with tryptic enzyme specificity. Average parent ion mass tolerance was 2.5 Da. Monoisotopic fragment ion mass tolerance was 1.0 Da. The ion series used in scoring were b and y. A static modification of +57 Da was added to all cysteine residues. A variable modification of +16 Da on methionine residues was also allowed.

We used a linear discriminant transformation to improve the identification sensitivity from the SEQUEST analysis (27, 28). SEQUEST DTA and OUT files were compressed using in-house Python scripts (PAW_MudPIT_Zipper.py, version 1.2). The zipped results files were converted to SQT and MS2 files (29), SEQUEST scores combined into linear discriminant function scores, and discriminant score histograms created separately for each peptide charge state (1+, 2+, and 3+), number of tryptic termini (0, 1, or 2), and modification state (unmodified or M+16 modified). Separate histograms were created for matches to forward sequences and for matches to reversed sequences (PAW_convert_3.1.py, version 3.1). The score histograms for reversed matches were used to estimate peptide false discovery rates (FDR) and set score thresholds for each peptide class that achieved the desired peptide FDR (typically 1% unless noted otherwise). Identifications not passing the score thresholds were removed from the SQT and MS2 files by another script (PAW_filter_mods.py, version 1.2). The entire set of confidently identified peptides for each species was collectively mapped to their respective protein databases. Proteins identified by identical sets of peptides were grouped together as redundant proteins. Proteins identified by a peptide set that was a formal subset of anther protein's peptide set were removed (parsimony principle). Proteins that were not identified by at least two distinct peptides having at least one-tryptic termini per sample were removed from the final list of identified proteins. Protein false discovery rates were estimated from decoy protein matches. Peptide-to-protein mapping and protein filtering were performed using PAW_results_6.py (version 6.1). The in-house Python scripts have been described previously (28). The identified peptides are supplied as supplemental material (supplemental Tables S4 and S5).

All the statistical analyses were performed using the spectral counts corrected for the shared peptides. The corrected spectral counts were obtained by the sum of unique counts plus some fraction (splitting) of shared peptide counts. The fraction is estimated as the relative proportion of unique peptides to total unique peptides for all of the proteins containing the shared peptide. To filter for false positive interactors, we compared the spectral counts of the main test samples (MLE-FLAG IP without any treatment) to the empty-vector samples and the GFP-FLAG samples treated as control replicates. The statistical validation was obtained through a beta-binomial test using the package developed by Pham *et al.* (30). Raw *p* values were then adjusted using the Benjamini-Hochberg method, false discovery rate (FDR) <0.2. MLE samples were normalized for the number of MLE protein spectral counts present in each condition (MSL2kd and RNase) and compared with the MSL2kd and the RNase samples to identify the protein affected by the different treatments. The β-binomial test with significance 0.05 was used for statistical validation. MLE samples were further corrected for differential gene expression under MSL2kd conditions using the data set produced by Zhang *et al.* (31).

##### Genome Analysis

Mi-2 (ID 926, 3675 and 3676), MLE (ID 3040 and 3788), JIL-1 (ID 945 and 3038) and MOF (ID 3044) ChIP-chip data sets were obtained from modENCODE and their peak files were analyzed using the Galaxy platform (http://galaxyproject.org). Files were filtered to retain only enriched regions and the resulting genomic intervals were then concatenated and merged to create a single data set for each protein. Mi-2 intervals coverage of MLE sites was calculated for every chromosome and the fraction of bases covered by each interval was used to draw box plots. Statistical relevance of the correlation among the data sets for sites on the second and third chromosome was determined by integrating the GenometriCorr package (32) into Galaxy. By intersecting Mi-2 and MLE regions on the second and third chromosomes we selected the intervals with an exact base pair overlap between the two data sets. In order to determine their distribution in the genome, the overlapping sites were analyzed with the CEAS package, available at the Cistrome installation on Galaxy (http://cistrome.org/ap/root). These regions were also mapped onto the genome-wide chromatin landscape of Kharchenko *et al.* (33), and their total base coverage of a particular distinct chromatin signature was used to calculate the percentage of this signature among Mi-2-MLE overlapping sites. The MOF total base coverage of enhancers and TSS regions overlapping with Mi-2-MLE sites was used to calculate the percentage of these intervals that is covered by MOF. The percentage of Mi2-MLE sites overlapping functional enhancers in S2 cells (34) was determined by calculating the number of sites intersecting the enhancers.

##### Drosophila melanogaster Culture

*D. melanogaster UAS-Pep* RNAi stock was obtained from the Bloomington stock center (# 32944), the *mle*[1] null allele was used in *mle* mutant analyses, and *mle[1]/Cy-OGFP larvae were used as wild type*. All stocks were maintained at 25 °C in vials that contained standard cornmeal/agar medium. Third instar larvae crawling along the vial walls were used for polytene chromosome preparations and Western blot analysis. To obtain uniformly aged larvae for qRT-PCR analysis, flies were raised on food containing 0.05% bromphenol blue (35) and third-instar larvae with dark guts were collected.

##### Polytenes Squashes and Immunostaining

Polytenes chromosome preparations and immunofluorescence staining were performed as previously described (36). Primary antibodies were used at the following concentrations: MSL1 (1:300), MSL3 (1:50), MLE (1:300), and the secondary antibody, Rodamin Red-X anti-Rabbit (Jackson IR, West Grove, PA) (1:500).

##### Immunoprecipitation and Western Blotting

FLAG-IPs were performed as in Cugusi *et al.* (23). For immunoprecipitations with endogenous proteins, 2 mg of S2 cell extracts were incubated with 2 to 10 μl of a specific antibody or generic anti-mouse IgG (Jackson IR), overnight at 4 °C. Protein G Agarose Beads (Millipore, Billerica, MA) pre-equilibrated in lysis buffer were then added for two hours at 4 °C. After washing four times in PBS buffer containing 1% Tween-20, the beads were resuspended in loading buffer. Samples were loaded in Criterion Tris-HCl gels (Bio-Rad, Hercules, GA) and transferred to a polyvinylidene difluoride membrane by using 10% methanol-Tris-glycine transfer buffer, following Bio-Rad's Criterion protocol. The blots were probed using antibodies against, MLE (1:3000), Hrb87F/P11 (1:200), PEP/X4 (1:200), Mi-2 (1:2000), MEP-1 (1:2000), p66 (1:1000), MSL1 (1:3000), Topo II (Santa Cruz Biotechnology T22C5 1:200), and with appropriate secondary antibodies conjugated to HRP. Detection was recorded on x-ray films by chemiluminescence using ECL Prime Western blotting detection reagent (Amersham Biosciences # RPN2232). MEP-1 and Mi-2 antibodies were a gift from C.P. Verrijzer, p66 antibody was a gift from R. Nusse, Hrb87F and PEP antibodies were a gift from H. Saumweber.

##### RNA Extraction and qRT-PCR Analysis

RNA was isolated from 7 to 10 larvae per sample using the Qiagen RNeasy mini-kit with on-column DNA digestion, following the manufacturer's protocols. Real-time, reverse transcription-PCR was performed using the iScript One-step RT-PCR kit with SYBR Green (BIO-RAD). Differential gene expression analysis was performed according to the method of Pfaffl (37). Standard curves for each primer set were obtained to calculate their pairing efficiency. Pka-C1, showing the most consistent expression among the housekeeping genes tested, was used to normalize transcription measurements. The results of three independent biological replicates were averaged. The primers used to detect the Eip74EF transcript are: forward 5′-GCTGCGGAACATATGGAATC-3′ and reverse 5′-TGCGTTGAAGTAGGACGTTG-3′. The primers used to detect Eip75B are: forward 5′-CTGCCAGTATTTCCAGTCGC-3′ and reverse 5′-CAATGTCCACCTGCAGTTCC-3′. The primers used to detect BR-C are: forward 5′-CGCATCCTTAGTTTCGGTGG-3′ and reverse 5′-GTGGTCGTTGTTGTGGTTGT-3′. All the other primers used are from Cugusi *et al.* (23).

## RESULTS

### 

#### 

##### MLE Interacts with a Variety of Factors Involved in Nucleic Acids Metabolism

In order to identify new interactors of MLE, we performed a series of immunoprecipitations from S2 cells expressing a FLAG-tagged MLE protein. Following elution with a FLAG-peptide, the eluates were analyzed by mass spectrometry (MS). Three different types of negative controls were produced: untransfected S2 cells extracts with added FLAG peptide, cells expressing an empty vector, and cells expressing a FLAG-GFP protein. In order to distinguish the interactions of MLE with unknown factors from its known interaction with the subunits of the MSL complex (7, 9, 10, 38) we obtained a FLAG-tagged MLE precipitate from S2 cells where the MSL complex was abrogated by RNA interference against the MSL2 subunit (MSL2kd). In addition, because MLE has two RNA-binding sites, a sample was treated with RNase prior to immunoprecipitation (RNase). A scheme of the experimental design can be found in supplemental Fig. S1. We conducted a pilot experiment using 6 mg of cell extracts and a limited number of samples to test our approach. The identification of proteins involved in RNA and DNA metabolism and a measurable effect of the different treatments used (MSL2kd and RNase) on some of the interactions (supplemental Table S1), encouraged us to proceed with the planned experiment. To improve the sensitivity of our analysis we increased the amount of cell extracts to 10 mg. A total of 929 Drosophila proteins were identified (supplemental Table S2). Because some of the potential interactors shared a high degree of homology, we chose to perform all the analyses on the spectral counts corrected for the shared peptides rather than on the unique spectral counts. The control sample originating from untransfected S2 cells contained an extremely low number of total spectral counts in comparison to the other samples, therefore we could not use this control for statistical purposes. The empty vector and the FLAG-GFP samples performed similarly, thus we decided to consider them as control replicates in a beta-binomial test. Using an FDR cutoff of <0.2, 140 MLE confident interactors were identified (supplemental Table S3). Normalized MLE sample spectral counts were then compared with MSL2kd and RNase samples (supplemental Table S3). The RNase treatment reduced the binding of 81 proteins, in agreement with previous observations that MLE binding to chromatin and other proteins is mediated by RNA (39). In absence of the MSL complex, the affinity of MLE for 42 proteins appeared to be affected. In order to test the possibility that this outcome may result from an indirect effect of MSL complex abrogation, we further adjusted the number of spectral counts for gene expression variations in cells were MSL2 had been knocked down (31). This approach revealed that only in 24 cases the interaction was significantly affected. The persistence of the majority of the interactions in the absence of the MSL complex indicates that MLE is involved in biological processes, other than dosage compensation, that are presumably present in both sexes. Although the subunits of the MSL complex usually co-precipitate with MLE (23, 40) none of them were found in the MS analysis, consistent with previous observations (41). To demonstrate such interactions by MS probably requires highly cross-linked chromatin as starting material (42). Proteins involved in a variety of biological processes were identified by the MS analysis however, in consideration of the known features of MLE, we decided to focus our attention on partners with a clear function in nucleic acid metabolism.

Among the 51 interactors with a clear function in nucleic acid metabolism (Table I), many are involved in RNA processing such as splicing factors, translation regulating factors and several RNA helicases. We also found proteins active in chromatin remodeling, such as the Mi-2 ATPase and the nucleosome assembly protein 1, and components of the DNA replication machinery. In order to validate the preliminary indications that MLE participates in biological processes other than dosage compensation, we selected a subset of interactors and further characterized their association with MLE.

**Table I:** MS-identified MLE interactors involved in nucleic acid metabolism

Protein description	Biological process	Effect of MSL2kd	Effect of RNase treatment
CG30122	Splicing	Decrease	Decrease
IGF-II mRNA-binding protein	Splicing		Decrease
No on or off transient A	Splicing		Decrease
polyA-binding protein	Splicing		Decrease
Srp54	Splicing		Decrease
SF2	Splicing		Decrease
Rm62	Splicing		
Quaking related 58E-1	Splicing		Decrease
Zinc-finger protein at 72D	Splicing	Decrease	Decrease
CG5641	Splicing		Decrease
CG9684	Splicing		Decrease
Lark	Splicing		Decrease
Rasputin	Splicing	Decrease	Decrease
CG11266	Splicing		Decrease
Lethal (3) 72Ab	Splicing		
Aly	Splicing		Decrease
CG7185	Alternative splicing		
Heterogeneous nuclear ribonucleoprotein at 87F	Alternative splicing	Decrease	Decrease
Heterogeneous nuclear ribonucleoprotein at 98DE	Alternative splicing		Decrease
Argonaute 2	RNAi		Decrease
CG8414	rRNA processing		
nop5	rRNA processing		Decrease
Fibrillarin	rRNA processing		Decrease
Sister-of-Sex-lethal	RNA binding		Decrease
CG1316	RNA binding		
CG13472	RNA binding		Decrease
Protein on ecdysone puffs	RNA binding		Decrease
Heterogeneous nuclear ribonucleoprotein at 27C	mRNA localization		Decrease
Glorund	mRNA localization	Decrease	Decrease
Belle	RNA helicase		Decrease
CG10777	RNA helicase	Increase	Decrease
CG5800	RNA helicase	Decrease	Decrease
CG7878	RNA helicase		
Upf1	mRNA-decay		Decrease
eIF-5A	Translation		
eIF3-S9	Translation		
Eukaryotic initiation factor 2beta	Translation		
Eukaryotic initiation factor 3 p66 subunit	Translation	Decrease	Decrease
Eukaryotic translation initiation factor 4G	Translation		
CG8636	Translation		
CG10990	Translation	Decrease	Decrease
CG6094	Translation		Decrease
Mi-2	Chromatin remodeling		
Nucleosome assembly protein 1	Chromatin remodeling		
Nucleoplasmin	Chromatin remodeling		
Minichromosome maintenance 7	DNA replication		
Minichromosome maintenance 5	DNA replication		
Replication factor C subunit 3	DNA replication		
Rat1	DNA catabolic process		
SMC2	Chromosome condensation		
Translationally controlled tumor protein	DNA repair		

##### MLE Associates with Members of the Spliceosome Complex

The spliceosome is a complex of numerous ribonucleoproteins and other proteins that is responsible for the splicing of primary transcripts. The Drosophila spliceosome has been characterized by immunopurification and MS, and among the over 100 listed components, MLE was not found (43). In contrast, our MS analysis revealed the association of MLE with 16 spliceosomal proteins and three proteins involved in alternative splicing. Notably Aly, associated with the exon junction complex (EJC) and SF2, an essential splicing factor active in multiple steps. As expected most of these interactions are not perturbed by the abrogation of the MSL complex although they appear to be significantly affected by the exposure to RNase. The interaction between MLE and the spliceosome factors appears to be robust as 6 proteins had been also identified in the MS pilot study.

##### MLE Interacts with Heterogeneous Nuclear Ribonucleoproteins Involved in RNA Processing and Heterochromatin Protein 1a (HP1a) Deposition

**Fig. 1.**
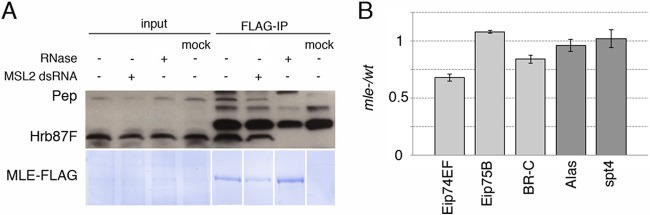
**MLE interacts with Hrb87F and Pep.** *A*, Aliquots from samples used in Fig. 2*A* were analyzed by Western blot with anti-Pep and anti-Hrb87F antibodies. Both proteins bind MLE regardless of the presence or absence of the MSL complex. Treatment with RNase A completely abrogates the binding. MLE-FLAG is stained with Coomassie. *B*, qRT-PCR showing the effect of an *mle* null mutation on the transcription of the ecdysone-responsive genes (light gray) *versus* ecdysone-unrelated genes (dark gray).

Three proteins, Pep (Protein on ecdysone puffs, CG6143), Hrb87F (heterogeneous nuclear ribonucleoprotein at 87F, CG12749) and Hrb98DE (heterogeneous nuclear ribonucleoprotein at 98DE, CG9983) were found to co-immunoprecipitate with MLE in an RNA-dependent manner. Pep and Hrb87F had been previously identified in the MS pilot study (supplemental Table S1). They have been shown to associate with HP1a, a protein that is involved in gene silencing and heterochromatin formation, as well as in the positive regulation of numerous euchromatic genes (44). Hrb98DE shares ∼80% of identity with Hrb87F. They are the Drosophila homologs of hnRNP A1 and appear to have redundant functions; they are mainly involved in the regulation of alternative mRNA splicing (43, 45). We confirmed the interaction of FLAG-MLE with Pep and Hrb87F by co-IP and Western blot analysis (Fig. 1*A*). These interactions are independent of the presence of the MSL complex and are abrogated by RNase treatment.

The loss of Hrb87F has no apparent effect on viability (46, 47), although some physiological functions appear to be affected (48). Because no similar information was available regarding Pep, we made use of a Drosophila line containing an inducible UAS-RNAi construct specific for the *Pep* gene that we induced with two different Actin-Gal4 drivers with ubiquitous expression. The results indicate that Pep abrogation reduces viability in both sexes and, although the knockdown efficiency is comparable in the two sexes, that males appear to be more severely affected (supplemental Fig. S2). Given the latter observation, we used immunofluorescence on polytene chromosomes to determine whether the distribution of the MSL complex was altered in PEP-deficient males. At the level of resolution afforded by this technique, we observed no disturbance in the distribution of the MSL complex (supplemental Fig. S3). We tested the expression of a small set of X-linked and autosomal genes. A general decrease of most of the transcripts was observed without a specific effect on the X (supplemental Fig. S4). We are led therefore to the conclusion that the increased loss of viability exhibited by Pep-deficient males is a reflection of a general, greater sensitivity of this sex to the genetic load.

In polytene chromosomes, Pep localizes at several euchromatic bands with a preference for ecdysone-induced puffs, where it appears to be associated with a hnRNP complex (49). MLE exhibits a similar pattern on autosomes in both sexes ((50) and our own unpublished data). These observations led us to ask whether MLE is involved in the ecdysone response. We measured the expression of three early ecdysone-responsive gene: Eip74EF, BR-C and Eip75B (51, 52). In order to avoid indirect effects because of MLE's function in dosage compensation, the analysis was performed in female larvae mutant for MLE. These females exhibit a 32% reduction in Eip74EF transcript and a 16% reduction in BR-C transcript, whereas Eip75B appeared to be slightly increased (Fig. 1*B*). Two genes not responsive to ecdysone and not bound by MLE (according to ChIP-chip data available on the modENCODE database) were also analyzed: Alas and spt4. These genes were expressed at a comparable level in mutant and wild type larvae. Ecdysone-responsive genes have been shown to exhibit a certain degree of heterogeneity in response to conditions affecting the pathway (52). The mild but consistent reduction observed in two of three ecdysone genes tested suggests a contribution of MLE in reaching wild type levels of transcripts during ecdysone induction.

##### MLE Co-immunoprecipitates with Subunits of the NuRD and dMEC Complexes

**Fig. 2.**
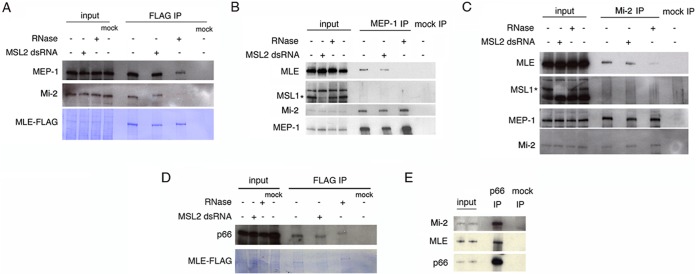
**MLE interacts with members of the NuRD complex.** *A*, MLE-FLAG immunoprecipitation from S2 cells extracts. MLE binds the MEP-1 and Mi-2 subunits of the NuRD complex; this binding is not affected by the presence of the MSL complex but is severely affected by RNase treatment. Mock samples are S2 cell extracts containing FLAG peptide. MLE-FLAG is stained with Coomassie. *B*, MEP-1 immunoprecipitation from S2 cell extracts. MEP-1 binds endogenous MLE whereas MSL1 does not appear to be present in the precipitate. Mi-2 was used as a positive control for the immunoprecipitation. Mock IP is performed with generic IgG. *C*, Mi2 immunoprecipitation from S2 cell extracts. Mi-2 binds its partner MEP-1 and MLE but not MSL1. MSL1 is reduced in the MSL2 knockdown samples because it is largely unstable without MSL2. Mock IP is performed with generic IgG. *D*, MLE-FLAG immunoprecipitation from S2 cell extracts. p66 binding to MLE does not require the MSL complex and is partially affected by RNase treatment. Mock samples are S2 cell extracts containing FLAG peptide. MLE-FLAG is stained with Coomassie. *E*, p66 immunoprecipitation from S2 cell extracts. p66 specifically interacts with MLE and with its known partner Mi-2. Mock IP is performed with generic IgG.

Mi-2, a subunit of the Drosophila NuRD complex (53) and of the dMEC complex (54) was identified in the MS analysis. In the pilot study, another component of these two complexes appeared to interact with MLE: MEP-1. We confirmed the binding to MLE-FLAG of both, Mi-2 and MEP-1, by Western blot analysis (Fig. 2*A*). In order to validate these interactions, we performed reverse immunoprecipitation experiments with untransfected S2 cells using antibodies against MEP-1 (Fig. 2*B*) and Mi-2 (Fig. 2*C*), respectively. In the first case, we noted the presence of MLE and of Mi-2 and in the second case, the presence of MLE and MEP-1. The level of interaction of these proteins appears to be significantly enhanced by the presence of RNA in the extract. MSL1 is not detectable in the immunoprecipitates and MSL2 knockdown does not alter the level of binding. These observations lead us to suggest that the MSL complex is not involved in this interaction. These results did not allow us to establish with which of the complexes (dMEC, NuRD or both) MLE can interact. Therefore we tested for the presence of p66, a subunit specific for the NuRD complex, in a FLAG-MLE immunoprecipitation (Fig. 2*D*). p66 behaves similarly to Mi-2 and MEP-1, its interaction with MLE does not require the MSL complex and it is reduced but still present after RNase treatment. We confirmed the association of MLE with p66 by performing a reverse IP with a p66-specific antibody (Fig. 2*E*) using untransfected S2 cells extracts. As expected (53), MEP-1 was also detectable in the precipitate (supplemental Fig. S5*A*). However, although both MEP-1 and Mi-2 co-precipitate when antibodies against either of these subunits are used, the presence of p66 is obvious only when anti-Mi-2 is used for immunoprecipitation (supplemental Fig. S5*B*). In the anti-MEP-1 precipitate, p66 is visible only after strong overexposure of the filter (data not shown). This difference may be explained by the observation that dMEC is the major Mi-2 complex during Drosophila embryogenesis (54) and S2 cells used in our experiment are of embryonic origin.

The results just described lead to the conclusion that MLE is a partner of the NuRD complex and also a possible component of the dMEC complex. As a chromatin remodeling complex, NuRD might act in conjunction with topoisomerases. In fact, Topo II was found in the same sucrose gradient fraction that contained the *Xenopus laevis* NuRD complex subunits (55), and it is a partner of MLE in the MSL complex (23). Therefore, we tested whether MEP-1 and Mi-2 co-immunoprecipitate with Topo II and found a robust binding of the topoisomerase to both proteins (supplemental Fig. S6*A* and S6*B*); this binding appears to increase in absence of the MSL complex and is completely disrupted after RNase treatment.

**Fig. 3.**
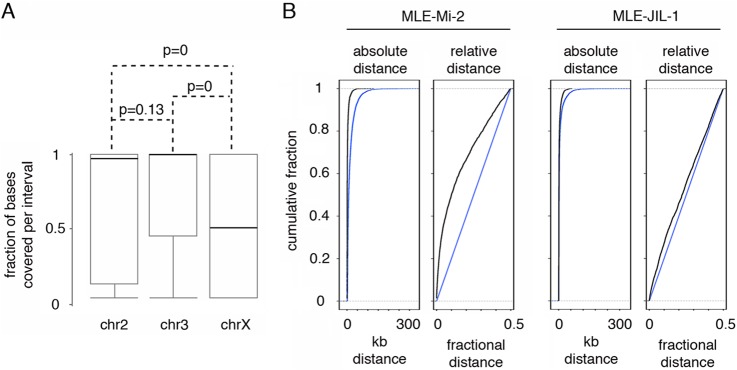
**MLE and Mi-2 ChIP-chip data analysis.** *A*, Box plot showing Mi-2 coverage of MLE enriched regions in S2 cells. The median Mi-2 coverage of MLE sites on the second (chr2) and third (chr3) chromosomes is ∼1, indicating that the majority of MLE intervals lies on Mi-2-enriched regions. In the X chromosome the median is ∼0.5, indicating a poor correlation between MLE and Mi-2, likely because of the prevalent association of MLE with the MSL complex on this chromosome. The black lines represent the medians. *B*, GenometriCorr analysis of MLE sites on the second and third chromosomes *versus* Mi-2 or JIL-1 sites. ECDF plots of absolute distance and relative distance tests show a greater correlation between MLE and Mi-2 interval midpoints than MLE and JIL-1. Blue lines represent the estimated distributions of uncorrelated data, black lines show the distributions of the experimental data.

**Fig. 4.**
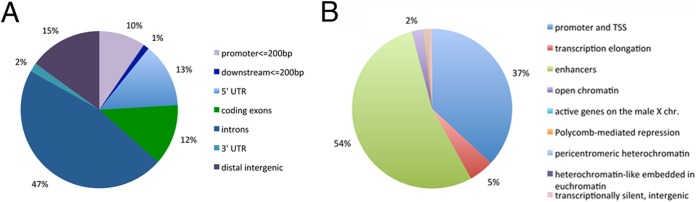
**Distribution of MLE and Mi-2 overlapping sites.** *A*, distribution of Mi-2 and MLE overlapping fragments on functional genomic regions (*B*) distribution of Mi-2 and MLE overlapping fragments on chromatin states.

We used S2 cells ChIP-chip data sets of Mi-2 and MLE, available from modENCODE, to compare their genomic localization. An analysis of Mi-2 coverage of MLE enriched sites revealed an impressive overlapping of the two proteins on the second and third chromosomes (Fig. 3*A*). As expected, overlap on the X chromosome was significantly less than that on the autosomes (Kolmogorov-Smirnov *p* value = 0) because on the X, because of its association with the MSL complex, MLE binds to numerous sites that do not necessarily correlate with Mi-2 localization. This finding suggests that MLE localizes at a subset of Mi-2 sites on the autosomes. To further validate the results obtained with chromosomes 2 and 3, we compared Mi-2 and MLE distributions using the Genometric Correlation package (32). Tests for the absolute and relative distance of interval midpoints (Fig. 3*B*) show a positive correlation (Kolmogorov-Smirnov *p* value = 0 and area permutation test *p* value <0.01); similarly Jaccard (*p* value < 0.01) and projection tests (*p* value = 0) reveal a significant level of overlap between the two data sets. Together these results indicate that Mi-2 and MLE tend to be very close or to overlap more frequently than expected on a random basis. It is possible that the positive correlation observed is because of the independent binding of both, MLE and Mi-2, over sites recruiting a broad group of proteins. In order to investigate this hypothesis, we analyzed the correlation between MLE and JIL-1, a chromatin kinase associated with active transcription that, according to the MS data, does not interact with MLE. The plots in Fig. 3*B* show that MLE has a substantially greater correlation with Mi-2 (relative ECDF area correlation = 0.39) than with JIL-1 (relative ECDF area correlation = 0.09) strengthening the idea of a concomitant recruitment of Mi-2 and MLE. Nevertheless we cannot rule out the possibility that other complexes interacting with MLE, might contribute to MLE's localization at sites were Mi-2 is also present. Interestingly, overlapping fragments are particularly enriched in non-coding regions (Fig. 4*A*), leading us to check the position of these fragments in relation to the state of the chromatin, using the data set from Kharchenko *et al.* (33). As shown in Fig. 4*B*, the vast majority of the sites are in regions with marks for enhancers or for transcription start sites. We tested the list of S2 cells functional enhancers identified by Arnold *et al.* (34) and found that 46% of MLE-Mi-2 sites intersect functional enhancers, in agreement with the results obtained using the chromatin state classification. It has been recently reported that H4k16ac and MOF (a H4k16 specific acetyltransferase) are enriched at a subset of enhancers in mouse embryonic stem cells (56, 57). Because MOF is a partner of MLE in the context of the MSL complex, we decided to measure the level of coverage of MLE-Mi-2 sites by MOF. We observed that MOF covers the majority of the sites in TSS regions and ∼60% of the enhancer regions bound by Mi-2 and MLE (supplemental Fig. S7). This result shows that while MOF is present at a substantial number of MLE-Mi-2 sites in enhancers, it is unlikely that it is responsible for MLE recruitment at those sites. The high level of overlapping found at the TSS is not surprising given the inclusion of MOF in the NSL complex that is present in these regions (58, 59).

## DISCUSSION

MLE has been identified in Drosophila, as a member of the dosage compensation MSL complex (13, 60). Although this complex is assembled only in males, MLE is present in female somatic cells. RNA helicase A, the almost perfect mammalian ortholog of MLE, has been implicated in a multiplicity of regulatory steps and pathways. This has prompted us to initiate a search for similar interactions involving MLE in Drosophila. To this end, we have carried out a mass spectrometry analysis of the proteins that co-immunoprecipitated with MLE in S2 cells. As proof-of-principle, we have selected a representative sample of proteins involved in nucleic acid metabolism and confirmed their association with MLE.

The spliceosome is a very large multiprotein/snRNRPs complex that is highly conserved between humans and Drosophila. Helicases play a critical role in spliceosome function where they are responsible for disrupting RNA-protein and RNA-RNA interactions during the course of the splicing process (61). In an extensive characterization of the two complexes, RNA helicase A was detected in the human spliceosome but MLE was not present in the Drosophila complex (43). We have demonstrated that MLE does in fact co-immunoprecipitate with several other known subunits of the Drosophila spliceosome and is most likely an integral member of this complex.

MLE interacts with three proteins - Pep, Hrb98DE and Hrb87F - that belong to different classes of heterogeneous nuclear ribonucleoproteins involved in RNA processing. In Drosophila, Hrb87F and Pep are found at many active sites within the genome, including the ecdysone-induced puffs where they interact with HP1a (heterochromatin protein 1a). A similar localization was observed also for MLE (50). HP1a is thought to associate directly with gene transcripts (62) and it is reasonable to propose that a helicase may facilitate this interaction by altering the RNAs' secondary structure. Supporting this suggestion is our observation that the absence of MLE affects the level of transcription of ecdysone-responsive genes.

NuRD is a complex that increases histone/nucleosome density not only at its target sequences throughout the genome, but is also responsible for changes in nucleosome organization at neighboring loci (63). As is often the case with chromatin remodeling complexes, NuRD and dMEC can be associated with gene repression or activation (53). Using the MBD3 subunit as a signal, NuRD was found on CpG-rich promoters, on gene bodies and on enhancers in different breast cancer lines (64), a distribution that closely resembles the distribution of MLE and Mi-2 overlapping sites. In Drosophila, the NuRD complex isolated from embryos contains nine subunits that include the histone deacetylase RPD3, the zinc-finger protein MEP-1 and the chromodomain-helicase-DNA-binding ATPase CHD4/Mi2 (53). MEP-1 and Mi-2 form also a separate complex: dMEC (54). The Mi-2 subunit, common to both complexes, is required for the expression of heat-shock genes (65), and plays a critical role in chromosome structure by affecting the function of cohesin (66). Given the apparent association of MLE with subunits of the NuRD and dMEC complexes, and given its genomic co-localization with Mi-2, it may be surprising that this protein was not identified when the two Drosophila complexes were purified (53, 54). In this regard, it is useful to note that the purification of dMEC using either Mi-2 or MEP-1 antibodies did not lead to the co-purification of RPD3, a subunit of the NuRD complex (54). The association of MLE with Mi-2, MEP-1 and p66 signals that it is a possible functional partner of the NuRD and dMEC complexes. In mammals, the NuRD complex is known to contain an ATP-dependent helicase CHD3/Mi-2α or CHD4/Mi-2β, suggesting targeted gene specificity. Whether Drosophila NuRD complexes exist with the CHD4/Mi-2 replaced by MLE remains to be established.

An important goal of this research was to demonstrate the value of using MLE as a model for the study of its human ortholog RHA. Following their synthesis, most if not all RNA molecules assume secondary structures, or tertiary structures if they associate with proteins. These structures can interfere with the RNAs' ultimate biological functions and must be actively modified, a role assumed most prominently by Superfamily 2 helicases, in particular by the DEAD box and the DEAH families (67). MLE and RHA contain all of the conserved motifs found in this superfamily. They are referred to as DEAH helicases because the sequence of their motif II is Asp-Glu-Ala-His. Their amino acid sequences (1262 aa for MLE and 1280 aa for RHA) are 49% identical and 86% similar. Not surprisingly, each of the two helicases reacts with polyclonal antisera prepared against the other (15). DEAH helicases are involved in transcription, pre-mRNA splicing, translation, ribosome biogenesis and mitochondrial RNA splicing. Specifically, RHA has been associated with transcription where it can mediate the association of the co-activator CBP with RNAPII (68), interact with the transcription factor NF-κB (69), bridge β-actin with RNAPII (70), and allow the binding of EGFR to an AT-rich sequence of the target genes' promoters (21). It also plays a role in the selection of pre-polyadenylation sites in pre-mRNAs (71), in the translation of special mRNAs (19, 72), and in RNA interference (16, 17) although this conclusion has been challenged (73). A number of experimental results provide the indication that MLE may perform similar functions. MLE regulates the transcription of the roX2 long non-coding RNA of the MSL complex by binding to an AT-rich region of the promoter (74); it appears to be involved in pre-mRNA processing by resolving the double-stranded RNA structure that is necessary for adenosine-to-inosine RNA editing of some messages (14). Our own results strongly suggest an involvement of MLE in the function of the spliceosome complex, in the RNAi pathway as well as in transcription regulation. Validating the usefulness of using MLE as model for the study of RHA is the association with the NURD and dMEC remodeling complexes that has not been reported for RHA.

## Supplementary Material

Supplemental Data

## Footnotes

^1^ The abbreviations used are:

MLE: maleless
MSL: male specific lethal
HP1a: heterochromatin protein 1a.
