# Supplementary material for: The Drosophila Helicase Maleless (MLE) is Implicated in Functions Distinct From its Role in Dosage Compensation
Source: Mol Cell Proteomics. 2015 Mar 16;14(6):1478–88. doi: 10.1074/mcp.M114.040667 (PMC4458714; doi:10.1074/mcp.M114.040667)
Supplement: Supplemental Data [file supp_14_6_1478__index.html]

The Drosophila Helicase Maleless (MLE) is Implicated in Functions Distinct From its Role in Dosage Compensation — Multiple Roles of MLE — Supplemental Data 

# The Drosophila Helicase Maleless (MLE) is Implicated in Functions Distinct From its Role in Dosage Compensation

## Supplemental Data

**Files in this Data Supplement:**

- Supplemental Figures
- Supplemental Table 1 - Supplemental Table 1
- Supplemental Table 2 - Supplemental Table 2
- Supplemental Table 3 - Supplemental Table 3
- Supplemental Table 4 - Supplemental Table 4
- Supplemental Table 5 - Supplemental Table 5
