# Supplementary material for: The Drosophila Helicase Maleless (MLE) is Implicated in Functions Distinct From its Role in Dosage Compensation
Source: Mol Cell Proteomics. 2015 Mar 16;14(6):1478–88. doi: 10.1074/mcp.M114.040667 (PMC4458714; doi:10.1074/mcp.M114.040667)
Supplement: Supplemental Data [file supp_M114.040667_Supplemental_figures_03172015.pdf]

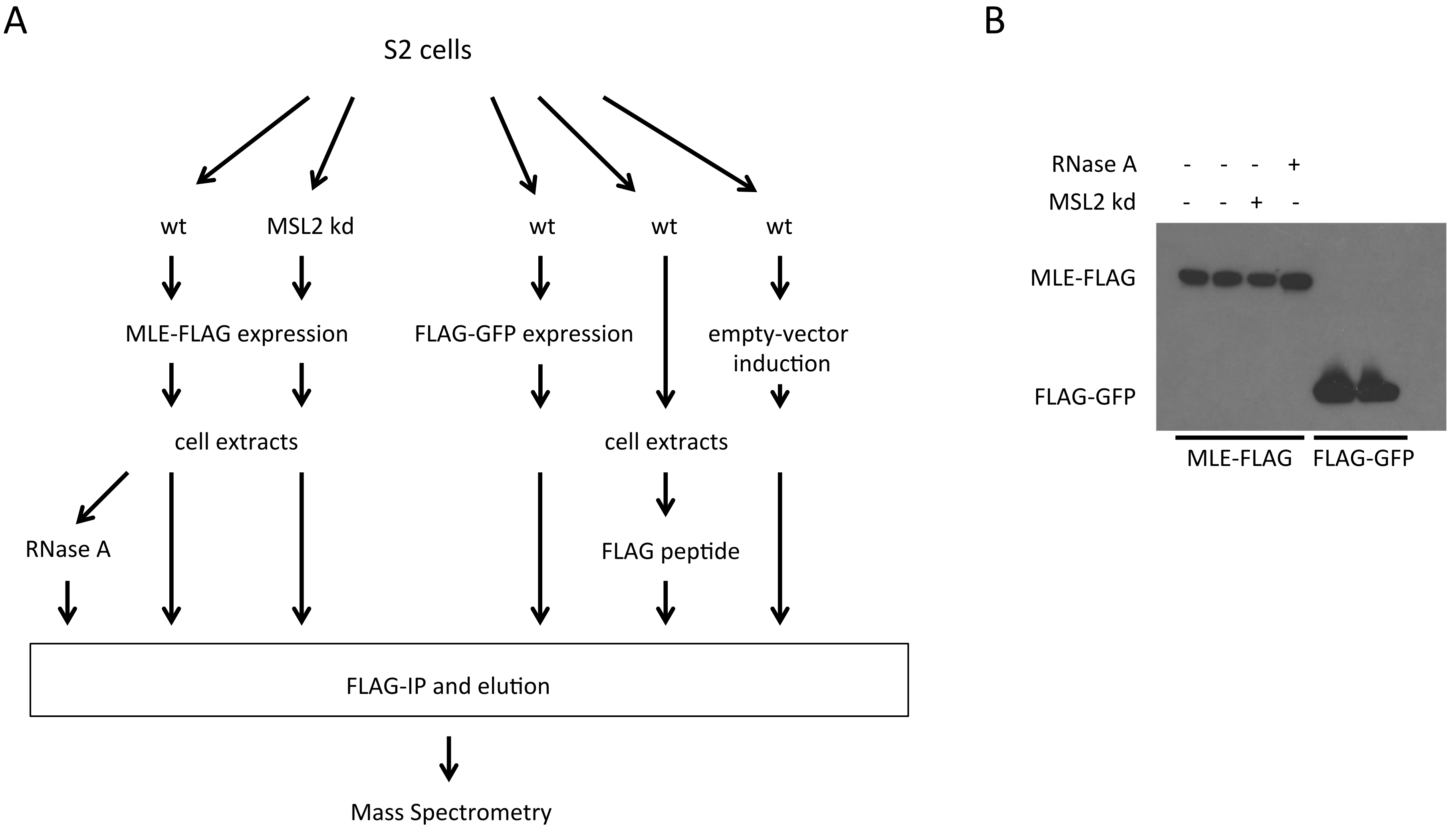

Supplemental figure 1: IP-MS. A) experimental design chart. B) Western Blot analysis of IP-eluates prior MS analysis.

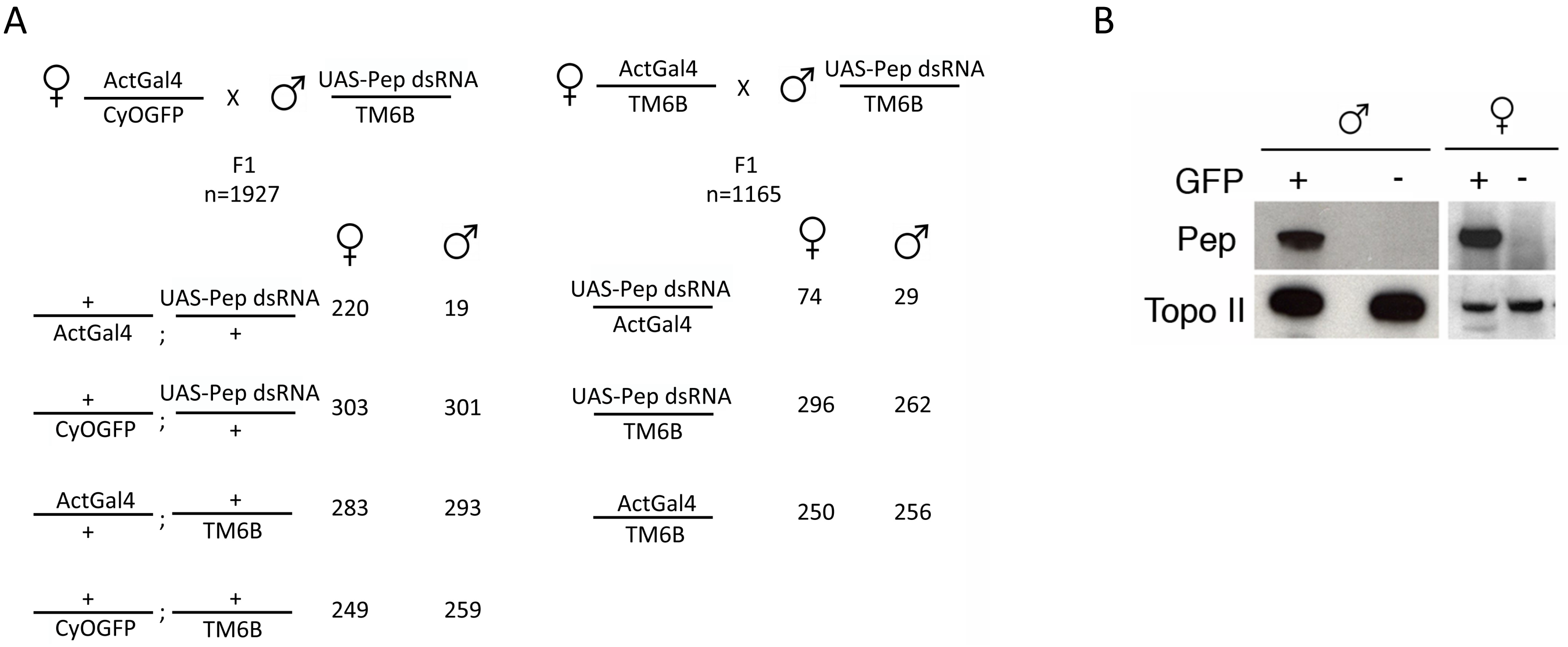

Supplemental figure 2: Pep knock down crosses. A) Crossing schemes. In crosses with an ActGal4 driver on the second chromosome (left), males appear to be more sensitive to Pep knock down. Using an ActGal4 driver on the third chromosome (right) we confirm the lethal effect of Pep knock down however the difference between males and females is less pronounced. B) Western blot analysis of Pep knock down. Brains and salivary glands from 30 larvae not tubby carrying either ActGal4 or CyOGFP on the second chromosome are tested for the presence of Pep, Topo II is used as a loading control. The knock down efficiency is comparable in males and females.

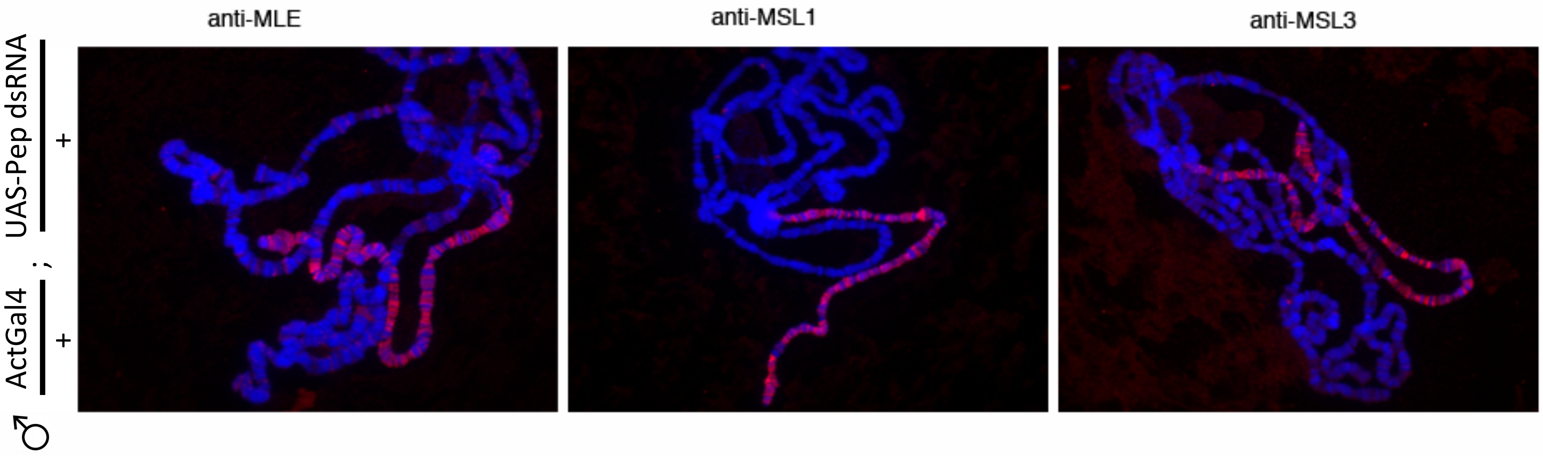

Supplemental figure 3: MSL complex localization on polytene chromosomes of males Pep knock down larvae. MSL complex subunits appear to be normally localized on the X chromosome. DAPI staining is in blue and the specific antibodies are in red.

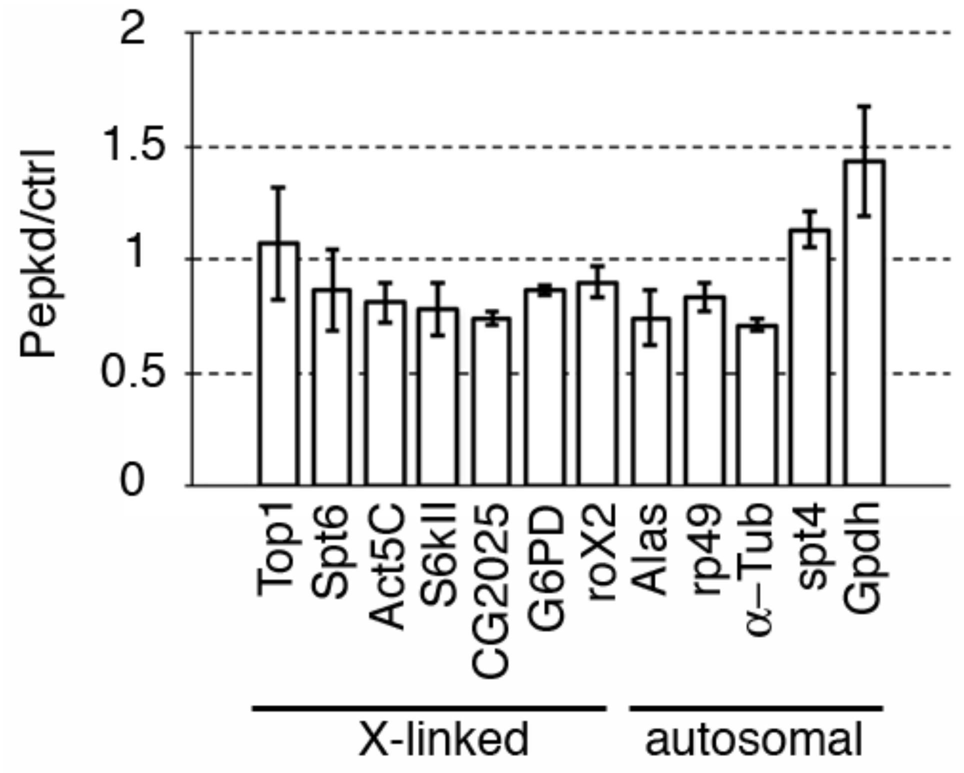

Supplemental figure 4: qRT-PCR from Pep knock down male larvae.
Differential gene expression analysis in larvae where Pep is knockdown compared to control larvae. Majority of the genes tested are down-regulated. The results are the average of three independent biological replicates.

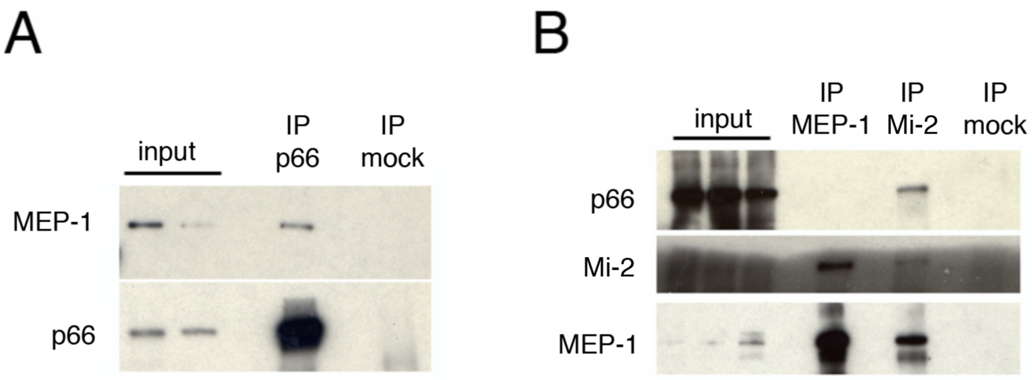

Supplemental figure 5: S2 cells extracts immunoprecipitations with subunits of the NuRD complex. (A) MEP-1 co-precipitates with p66. (B) p66 is efficiently co-precipitated by Mi-2 but not by MEP-1. Mock IPs were performed with generic anti-mouse IgG.

A

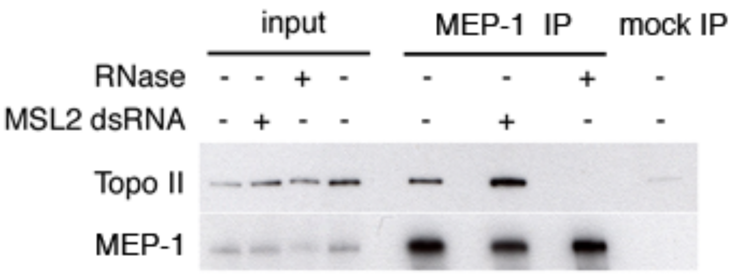

B

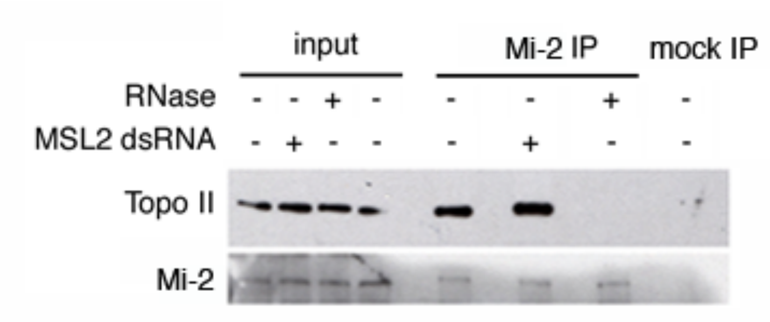

Supplemental figure 6: S2 cells extracts immunoprecipitations with subunits of the NuRD complex. A) MEP-1 binds Topo II in an RNA dependent manner. The MSL complex is not required for the interaction. B) Mi-2 interacts with Topo II, this interaction is disrupted by RNAse treatment and it is independent of the MSL complex. Mock samples are S2 cells extracts incubated with generic IgG.

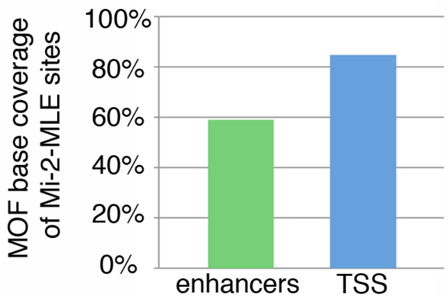

Supplemental figure 7: MOF coverage of Mi-2-MLE overlapping sites at enhancers and TSS.
